# Fungus-Derived 3-Hydroxyterphenyllin and Candidusin A Ameliorate Palmitic Acid-Induced Human Podocyte Injury via Anti-Oxidative and Anti-Apoptotic Mechanisms

**DOI:** 10.3390/molecules27072109

**Published:** 2022-03-25

**Authors:** Suchada Kaewin, Karn Changsorn, Titiwat Sungkaworn, Peraya Hiranmartsuwan, Wiriya Yaosanit, Vatcharin Rukachaisirikul, Chatchai Muanprasat

**Affiliations:** 1 Department of Physiology, Faculty of Science, Mahidol University, Rama VI Road, Ratchathewi, Bangkok 10400, Thailand; suchada.kaewin@gmail.com; 2 Chakri Naruebodindra Medical Institute, Faculty of Medicine Ramathibodi Hospital, Mahidol University, Bang Phli, Samut Prakarn 10540, Thailand; changsorn.k@gmail.com (K.C.); titiwat.sun@mahidol.edu (T.S.); 3 Department of Chemistry, Faculty of Science and Technology, Thammasat University, Khlong Luang, Pathumthani 12120, Thailand; peraya.ph@gmail.com; 4 Division of Physical Science and Center of Excellence for Innovation in Chemistry, Faculty of Science, Prince of Songkla University, Hat Yai, Songkhla 90110, Thailand; wiriya.ysn@gmail.com (W.Y.); vatcharin.r@psu.ac.th (V.R.)

**Keywords:** podocytes, diabetic nephropathy, 3-hydroxyterphenyllin, candidusin A, palmitic acid

## Abstract

Diabetic nephropathy (DN) is a leading cause of end-stage renal disease. An elevated fatty acid plasma concentration leads to podocyte injury and DN progression. This study aimed to identify and characterize cellular mechanisms of natural compounds that inhibit palmitic acid (PA)–induced human podocyte injury. By screening 355 natural compounds using a cell viability assay, 3-hydroxyterphenyllin (3-HT) and candidusin A (CDA), isolated from the marine-derived fungus *Aspergillus candidus* PSU-AMF169, were found to protect against PA-induced podocyte injury, with half-maximal inhibitory concentrations (IC_50_) of ~16 and ~18 µM, respectively. Flow cytometry revealed that 3-HT and CDA suppressed PA-induced podocyte apoptosis. Importantly, CDA significantly prevented PA-induced podocyte barrier impairment as determined by 70 kDa dextran flux. Reactive oxygen species (ROS) and 2,2-diphenyl-1-picrylhydrazyl (DPPH) direct scavenging assays indicated that both compounds exerted an anti-oxidative effect via direct free radical–scavenging activity. Moreover, 3-HT and CDA upregulated the anti-apoptotic Bcl2 protein. In conclusion, 3-HT and CDA represent fungus-derived bioactive compounds that have a novel protective effect on PA-induced human podocyte apoptosis via mechanisms involving free radical scavenging and Bcl2 upregulation.

## 1. Introduction

Diabetes mellitus (DM) has a major impact on the global population, with an estimated prevalence of 9.3% and a total of 463 million cases [1]. As a common and serious complication of DM, diabetic nephropathy (DN) develops in 20–40% of patients with DM, which could lead to end-stage renal disease (ESRD), requiring lifelong and costly interventions including dialysis and kidney transplantation [2,3,4]. Current treatments of DN are persistent control of blood glucose and blood pressure, which are of limited efficacy if they are started after symptom onset [5,6]. Therefore, there is an urgent need to develop novel effective therapeutic interventions that could effectively delay DN progression.

Type 2 DM (T2DM) has been found to be associated with elevated cellular oxidative stress [7,8]. Hyperglycemia, inflammation, and hyperlipidemia are important factors contributing to ROS production in T2DM through various signaling pathways, including nuclear factor kappa B (NF-κB) and protein kinase C (PKC) [7]. Intracellular reactive oxygen species (ROS) accumulation plays a crucial role in causing damage to major cellular components including lipids, proteins, and DNA in various organs and tissues [8]. Compounds with anti-oxidative capacity have been shown to ameliorate T2DM and its complications [9,10].

Podocytes play an essential role in maintaining glomerular filtration barrier integrity. Excessive production of oxidative stress under diabetic conditions leads to podocyte injury [11,12,13]. Podocyte injury and podocyte loss lead to progressive proteinuria, glomerulosclerosis, and eventually loss of renal function [14,15,16,17]. Previous studies have demonstrated that compounds with anti-oxidative activity could protect against podocyte injury and DN progression in vitro and in vivo [18,19].

The plasma free fatty acid (FFA) concentration is between 200 and 600 μM in healthy adults and could increase by up to 4-fold in patients with T2DM [20]. Accumulation of FFAs in podocytes is known to induce podocyte injury and death [20,21]. As the most abundant long-chain saturated FFA in the plasma, palmitic acid (PA) plays a crucial role in the pathogenesis of DN via mechanisms involving generation of ROS, resulting in cell damage and downregulation of Bcl2, a protein involved in anti-apoptotic signaling in many cell types including podocytes [22,23]. The prevention of PA-induced podocyte injury and podocyte death is considered to be a promising therapeutic approach for DN treatment.

Plants, microorganisms, and fungi are rich sources of bioactive compounds, which serve as potential candidates for development of new drugs. However, there is limited information on bioactive compounds against DN, especially those that prevent PA-induced podocyte injury. Therefore, the aim of this study was to identify compounds that inhibit PA-induced podocyte injury from a library of natural compounds isolated from plants and fungi in Thailand and to investigate their cellular mechanisms by using human podocytes.

## 2. Results

### 2.1. Establishment of an Experimental Model of PA-Induced Podocyte Death

**Figure 1:**
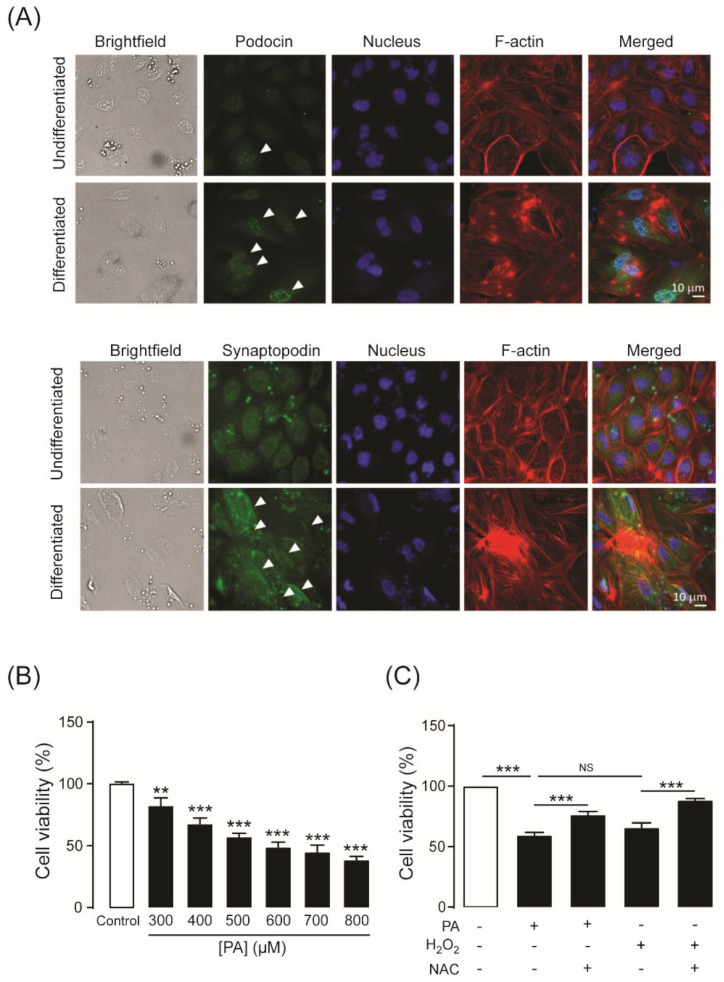
(**A**) Expression of podocyte-specific markers. Undifferentiated podocytes (upper panels) were cultured at 33 °C (permissive temperature). Differentiated podocytes (lower panels) were cultured at 37 °C (non-permissive temperature) for 14 days. Representative immunofluorescence images for podocin (green dots; arrows) and synaptopodin (green line; arrows) are shown. Podocin and synaptopodin were stained with an anti-podocin antibody and an anti-synaptopodin antibody, respectively. The nucleus was stained with Hoechst 33342 (blue color). F-actin filaments were stained with Alexa Fluor^TM^ 568 phalloidin (with the appearance of red lines). (**B**) Concentration-dependent response of palmitic acid (PA)–induced podocyte death. Podocytes were cultured in RPMI 1640 medium containing PA at concentrations between 300 and 800 µM for 24 h. Podocytes were incubated with 600 µM PA or hydrogen peroxide (H_2_O_2_) for 24 h. *N*-acetylcysteine (NAC) (10 mM), a glutathione precursor, was added with PA or H_2_O_2_. (**C**) Effect of PA on podocyte viability and mechanism of PA-induced podocyte death. Cell viability was measured with the MTT assay. The data are expressed as the mean ± standard error of the mean (*n* = 3–6). NS, non-significant; ** *p* < 0.01; *** *p* < 0.001, compared with the control or indicated group.

To determine whether podocytes were differentiated successfully into mature podocytes after culture at a non-permissive temperature (37 °C) for 14 days, the specific markers of differentiated podocytes including podocin and synaptopodin, were investigated by immunofluorescence staining. Nuclear localization of podocins was more intense in podocytes cultured under non-permissive conditions than cells cultured under permissive conditions (33 °C). Likewise, synaptopodin expression was found predominantly under non-permissive conditions, and it colocalized with the F-actin cytoskeleton (Figure 1A). This result indicates that the cells are differentiated into mature podocytes under non-permissive conditions.

Elevation of plasma FFAs is regarded as a crucial pathogenic factor in the development and progression of DN [24]. To establish a model of PA-induced podocyte death, podocytes were cultured for 24 h in RPMI-1640 medium containing PA from 300 to 800 µM. As shown in Figure 1B, cell viability decreased after PA treatment in a concentration-dependent manner. Since the plasma concentration of PA in patients with T2DM is ~600 µM, all subsequent experiments were evaluated at this concentration. We investigated whether oxidative stress was involved in the PA-induced podocyte damage by co-treatment with *N*-acetylcysteine (NAC), a well-known antioxidant. PA reduced podocyte viability to a similar extent as hydrogen peroxide (H_2_O_2_). Moreover, PA- and H_2_O_2_-induced podocyte damage was ameliorated significantly by co-treatment with NAC (10 mM) (Figure 1C). These results suggest that oxidative stress is a major contributor to PA-induced podocyte damage in this model.

### 2.2. Identification of 3-Hydroxyterphenyllin (3-HT) and Candidusin A (CDA) as Inhibitors of PA-Induced Podocyte Injury

**Figure 2:**
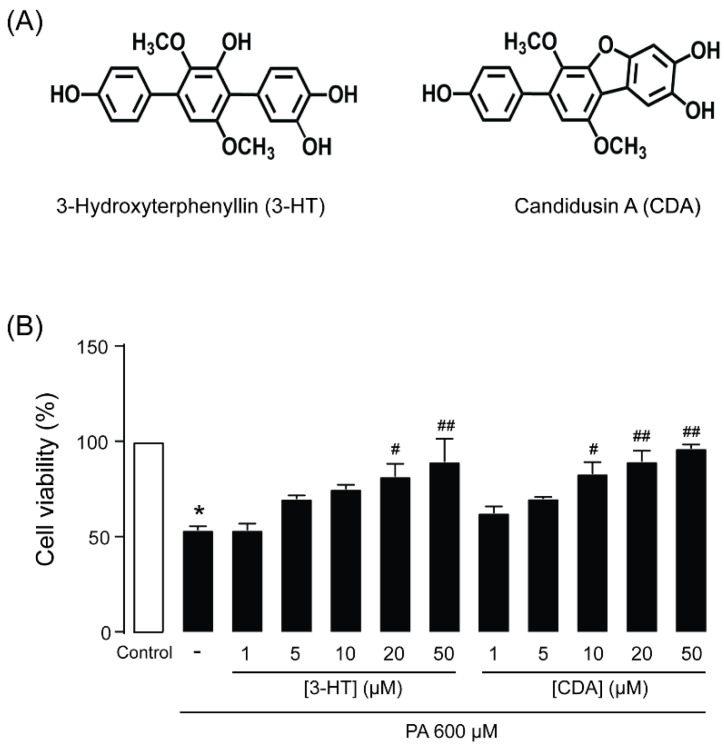
(**A**) The chemical structure of fungus-derived 3-hydroxyterphenyllin (3-HT) and candidusin A (CDA). (**B**) 3-HT and CDA protected against palmitic acid (PA)–induced podocyte death. Podocytes were cultured in RPMI 1640 medium containing 600 µM PA in the presence of 3-HT or CDA (1–50 µM) for 24 h. Cell viability was assessed with the MTT assay. The data are expressed as the mean ± standard error of the mean (*n* = 4). * *p* < 0.05 compared with control; # *p* < 0.05 compared with PA; ## *p* < 0.01 compared with PA.

To identify compounds capable of suppressing PA-induced podocyte injury, podocytes were cultured for 24 h in RPMI-1640 medium containing 600 µM PA with or without (control) test compounds (10 µM) before cell viability measurements using the MTT assay. Three hundred and fifty-five compounds isolated from fungi and plants listed in Table 1 were tested. We found that 3-HT and CDA (Figure 2A), isolated from the marine-derived fungus *Aspergillus candidus* PSU-AMF169, protected against PA-induced podocyte injury, increasing cell viability by 20% and 18%, respectively. Moreover, 3-HT and CDA inhibited PA-induced podocyte injury in a concentration-dependent manner, with half-maximal inhibitory concentrations (IC_50_) of 16.02 ± 6.05 and 18.37 ± 6.34 µM, respectively (Figure 2B).

### 2.3. Cytotoxicity of 3-HT and CDA in Human Podocytes

**Figure 3:**
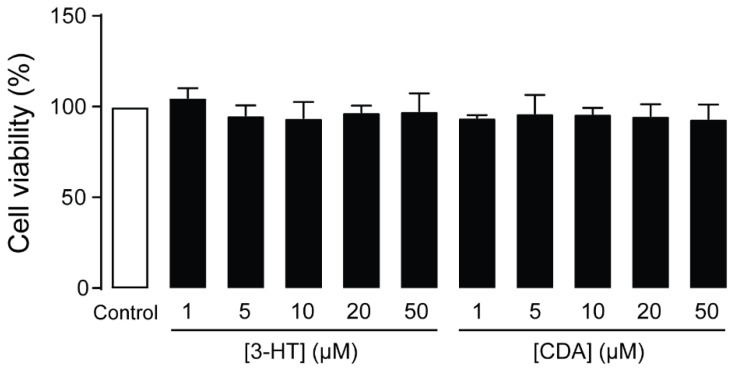
Cytotoxic evaluation of 3-hydroxyterphenyllin (3-HT) and candidusin A (CDA). Podocytes were grown on 96-well plates for 24 h with vehicle (control) or 3-HT or CDA at the indicated concentration before performing the MTT assay. The data are expressed as percent cell viability of control (*n* = 3). There was no significant difference between the control and treatment groups.

To investigate the potential cytotoxicity of 3-HT and CDA, the MTT assay was performed in human podocytes after 24-h exposure to these compounds at concentrations of 1–50 μM. These concentrations did not affect cell viability (Figure 3). Since both compounds demonstrated the greatest effect on suppressing PA-induced podocyte injury at 50 µM without affecting cell viability, this concentration was evaluated in the subsequent experiments.

### 2.4. Effect of 3-HT and CDA on PA-Induced Podocyte Apoptosis

**Figure 4:**
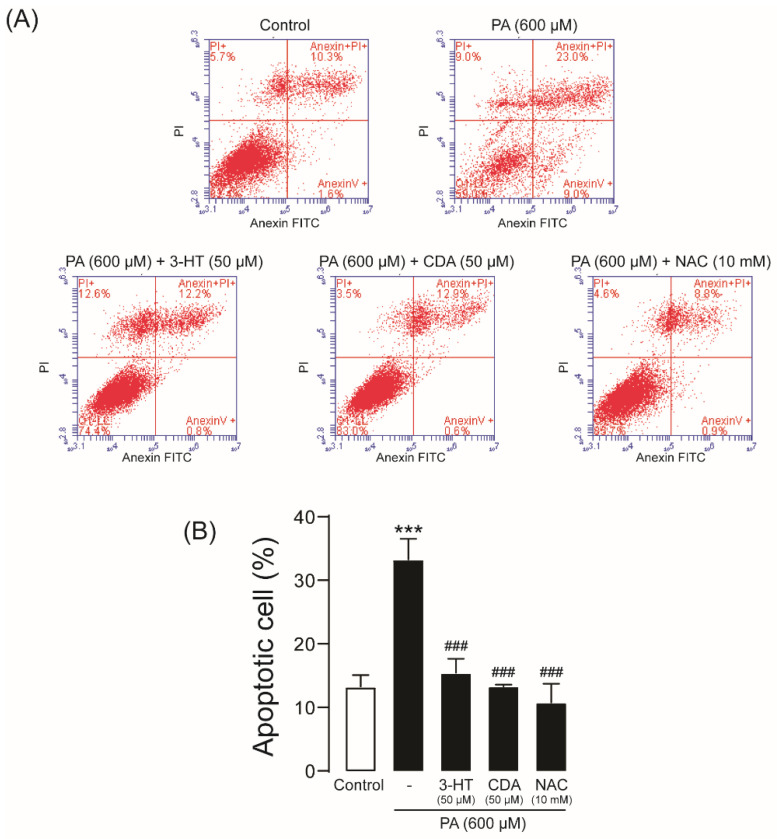
3-Hydroxyterphenyllin (3-HT) and candidusin A (CDA) prevent palmitic acid (PA)–induced podocyte apoptosis. Podocytes were cultured in 6-well plates and then incubated with 600 µM PA in the presence or absence of 50 µM 3-HT or CDA for 24 h. *N*-acetylcysteine (NAC) (10 mM) was used as a positive control. Annexin V/propidium iodide (PI) staining was performed. (**A**) The representative results of five independent experiments are shown. (**B**) Summary of the results. *** *p <* 0.001 compared with the control group; ### *p < * 0.001 compared with the PA-treated group.

To investigate the effect of 3-HT and CDA on PA-induced podocyte apoptosis, flow cytometric analysis was performed after annexin V/propidium iodide (PI) staining. PA treatment (600 µM) significantly increased the percentage of apoptotic cells (33.27% ± 1.88%) compared with control (13.27% ± 1.07%). Interestingly, co-treatment with 3-HT, CDA, or NAC almost completely inhibited PA-induced podocyte apoptosis, to 13.90% ± 1.55%, 13.30% ± 0.20%, and 10.77% ± 1.70%, respectively (Figure 4). This result suggests that 3-HT and CDA protect against PA-induced podocyte injury by suppressing apoptotic cell death.

### 2.5. Effect of 3-HT and CDA on PA-Induced Podocyte Barrier Leakage

**Figure 5:**
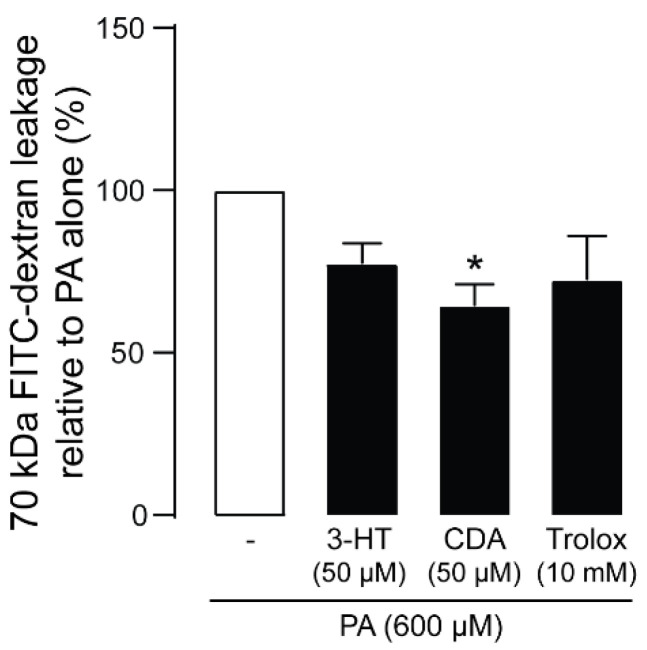
Effect of 3-hydroxyterphenyllin (3-HT) and candidusin A (CDA) on fluorescein isothiocyanate (FITC)–dextran leakage across the human podocyte monolayer. Podocytes were plated on a porous membrane and grown until confluence. Podocytes were treated with palmitic acid (PA) for 24 h with or without 50 µM 3-HT or CDA. Trolox (10 mM) was used as a positive control. FITC-dextran was added to the lower compartment, and its concentration in the upper compartment was measured after 120 min as an indicator of podocyte barrier leakage. The data are expressed as the mean of FITC-dextran concentrations relative to control (PA-treated) group ± standard error of the mean. * p < 0.05 compared with the PA-treated group.

Podocytes form a glomerular filtration barrier that impedes the leakage of plasma proteins into urine filtrate. We next investigated whether 3-HT or CDA prevented PA-induced podocyte barrier dysfunction by measuring fluorescein isothiocyanate (FITC)–dextran (70 kDa) leakage across the human podocyte monolayer. As shown in Figure 5, PA treatment impaired filtration barrier function, resulting in increased FITC-dextran flux, which was prevented by both compounds (50 µM) as well as Trolox (a positive control). CDA exerted this effect more potently than 3-HT.

### 2.6. Anti-Oxidative Mechanisms of 3-HT and CDA

**Figure 6:**
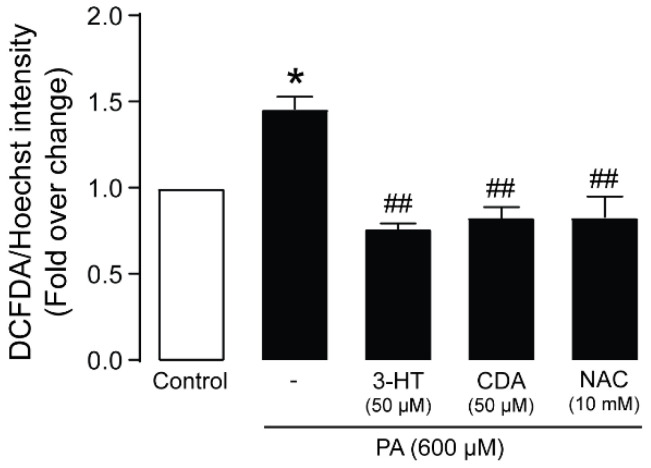
3-Hydroxyterphenyllin (3-HT) and candidusin A (CDA) attenuate reactive oxygen species (ROS) generation induced by palmitic acid (PA) in human podocytes. The level of ROS formation was measured by using the 2′,7′-dichlorofluorescin diacetate (DCFDA) fluorescent dye. Podocytes were seeded on 96-well black plates for 24 h before treatment with 600 µM PA and 3-HT or CDA at 50 µM, plus a vehicle (control) for another 24 h. *N*-acetylcysteine (NAC) (10 mM) was used as the positive control. The data are expressed as the mean intensity of DCFDA fluorescence/Hoechst ± standard error of the mean (*n* = 5). * *p* < 0.05 compared with the control group; ## *p* < 0.01 compared with the PA-treated group.

Since oxidative stress is a major contributor to PA-induced podocyte death, we investigated the effects of 3-HT and CDA on ROS generation in podocytes by using the 2′,7′-dichlorofluorescin diacetate (DCFDA) assay. As shown in Figure 6, 24-h treatment with PA increased DCFDA fluorescence intensity, indicating increased ROS generation, which was abolished by co-treatment with 50 µM 3-HT or CDA. NAC (10 mM) was used as a positive control. These data indicate that 3-HT and CDA protect against PA-induced podocyte death via anti-oxidative mechanisms.

**Figure 7:**
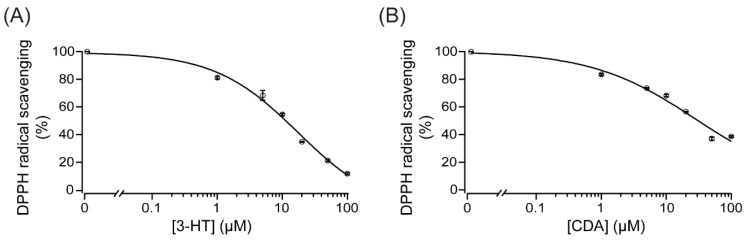
Direct reactive oxygen species (ROS) scavenging activity of 3-hydroxyterphenyllin (3-HT) (**A**) and candidusin A (CDA) (**B**). The antioxidant potential of 3-HT and CDA was evaluated by using the 2,2-diphenyl-1-picrylhydrazyl (DPPH) assay. DPPH was dissolved in 99% ethanol. Concentrations of 3-HT or CDA between 1 and 100 µM were tested. The absorbance of the solution was measured at 517 nm. A summary of the data fit to Hill’s equation is shown (*n* = 3).

We next investigated whether these compounds directly scavenge ROS by using the 2,2-diphenyl-1-picrylhydrazyl (DPPH) assay. As shown in Figure 7, 3-HT and CDA (1–100 µM) dose-dependently scavenged DPPH radicals, with IC_50_ of ~18 and 28 µM, respectively. These results suggest that both 3-HT and CDA exert their anti-oxidative effect, at least in part, by directly scavenging free radicals.

### 2.7. Effect of 3-HT and CDA on Apoptotic Signaling Proteins 

**Figure 8:**
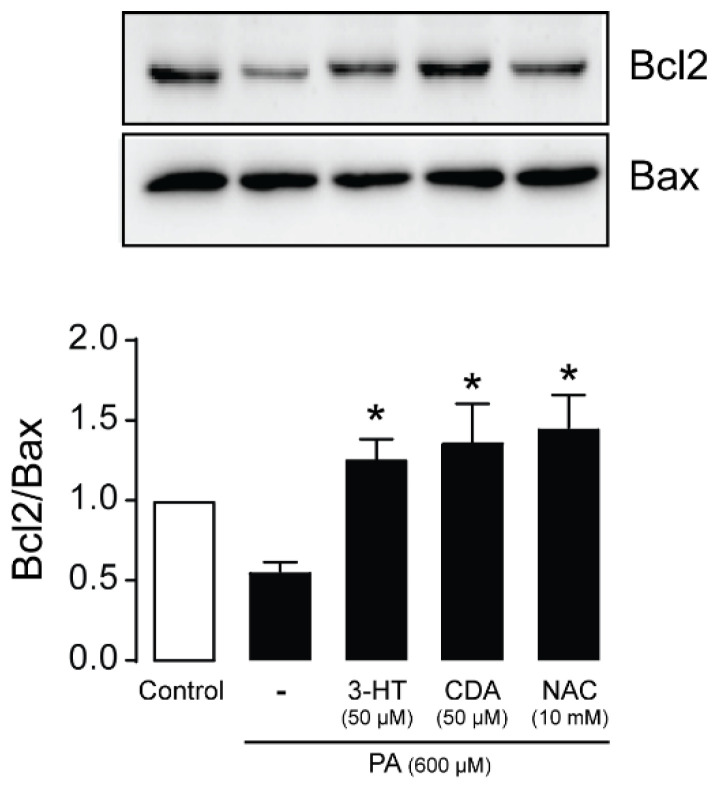
3-Hydroxyterphenyllin (3-HT) and candidusin A (CDA) upregulate the anti-apoptotic Bcl-2 protein. Podocytes were cultured in 6-well plates and then incubated with 600 µM palmitic acid (PA) in the presence or absence of 50 µM 3-HT or CDA for 24 h. *N*-acetylcysteine (NAC) (10 mM) was used as a positive control. Western blot analysis was performed (*n* = 6). The data are expressed as the band intensity of Bcl2/Bax (*n* = 6). * p < 0.05 compared with the PA-treated group.

Excessive ROS generation initiates apoptotic pathways by decreasing the protein expression ratio of the antiapoptotic Bcl-2 to the proapoptotic Bax [25]. We investigated the effects of 3-HT and CDA (50 µM) on the Bcl-2/Bax ratio in podocytes exposed to PA (600 µM) for 24 h. NAC (10 mM) was used as a positive control. As shown in Figure 8, treatment with PA reduced the Bcl2/Bax ratio; this reduction was completely reversed by co-treatment with 3-HT, CDA, or NAC.

## 3. Discussion

Elevation of plasma FFAs, especially PA, is one of the risk factors of DN development [22,26,27]. Excessive lipid accumulation in podocytes causes podocyte dysfunction or death [12]. Since a podocyte is a terminally differentiated cell with a limited capacity to proliferate, permanent loss of podocytes with no replacement will lead to loss of the stability and integrity of the glomerular filtration barrier. Therefore, podocyte death followed by albuminuria is an initial trigger of DN [28]. Based on this knowledge, identifying the compound(s) that specifically prevent(s) podocyte death may be a promising strategy for DN treatment. In this study, we have demonstrated for the first time that fungus-derived 3-HT and CDA could protect against PA-induced podocyte apoptosis via their anti-oxidative properties and upregulate the anti-apoptotic Bcl2 protein. The cytoprotective effect of 3-HT and CDA preserve the integrity of the glomerular filtration barrier, as seen by the reduction in FITC-dextran leakage.

In a normal subject, the total plasma FFA concentration has been reported as approximately 200–600 μM. However, the level of plasma FFAs increases up to 4-fold in patients with T2DM [20]. Elevation of systemic FFAs causes FFA deposition not only in adipocytes, but also in podocytes. Accumulation of FFAs in podocytes triggers podocyte injury or dysfunction. Palmitate is one of the primary circulating FFAs responsible for about 25% of the total FFAs [20]. Based on previous studies, PA shows a concentration-dependent cytotoxic effect in cultured mouse podocytes [20,26,29]. Consistently, in this study, PA (300–800 µM) dose-dependently reduced human podocyte viability after 24-h incubation. However, 600 µM PA was used in the subsequent experiments because this concentration correlates with the plasma PA level found in patients with T2DM.

Previous studies have reported that PA uptake into podocytes occurs via fatty acid transporter (FAT/CD36) [30]. Accumulation of PA in podocytes results in elevated ROS, changes in mitochondrial membrane potential, ATP depletion, and activation of apoptotic pathways [20,31]. In this study, incubation with 600 µM PA significantly decreased podocyte viability. In contrast, this cytotoxicity was almost completely abolished when co-treated with NAC. These data imply that oxidative stress is the major contributor to PA-induced human podocyte death [20,31].

Importantly, we found that 3-HT and CDA isolated from the marine-derived fungus *A. candidus* PSU-AMF169 protected against PA-induced podocyte death. Typically, the IC_50_ of a candidate therapeutic compound should be <10 µM. However, in this study, both 3-HT and CDA at 10 µM did not significantly reduce podocyte death. Therefore, we chose the concentration of 50 µM based on the experimental results: this concentration produced the maximum effect. Hence, at 50 µM, we could clearly observe an alteration in phenotypes. In addition, we demonstrated that neither 3-HT nor CDA affected cell viability at concentrations up to 50 µM. Similarly, both compounds isolated showed neither cytotoxicity toward human intestine cells (INT 407) nor mutagenicity toward *Salmonella typhimurium* at concentrations up to 100 µg/mL [32]. In contrast, 2–8 µM 3-HT significantly inhibits proliferation of human ovarian cancer cell lines, including A2780/CP70 and OVCAR-3 cells, by causing DNA damage, ROS accumulation, cell cycle arrest, and mitochondrial-driven cell apoptosis [33]. This discrepancy suggests that the cytotoxic effects of these compounds might be cell-type specific [34].

Before 3-HT or CDA can be translated to clinical use, they require structural modification. In future studies, we aim to test the IC_50_ of analogs to reveal the structure–activity relationship (SAR). This information may prove valuable to modify the parent compound to a more potent form with an IC_50_ < 10 µM. To investigate in vivo efficacy of these classes of compounds in the prevention of DN, modified compounds with favorable pharmacological properties, including high potency and low cytotoxicity, will be evaluated in rodent models of high fat diet (HFD)–induced DN in comparison and in combination with other anti-diabetic/anti-DN drugs.

Since oxidative stress is the major contributor to PA-induced human podocyte death, we hypothesized that 3-HT and CDA prevent podocytes from damage through their anti-oxidative properties. In general, antioxidants act via two main pathways. Some antioxidants directly scavenge ROS. On the other hand, some induce the Keap1–Nrf2 pathway, resulting in an increased anti-oxidative capacity [35,36,37]. Here, we demonstrated that 3-HT and CDA directly scavenged ROS: they counteracted DPPH radicals. Since both compounds contain numerous hydroxyl groups, they could donate their electrons to neutralize DPPH radicals. Moreover, 3-HT and CDA significantly reduced PA-induced podocyte ROS generation. This result is in agreement with a previous report showing that 3-HT could protect against oxidative damage to intestinal cells, and this protection was associated with its ability to reduce ROS formation and increase catalase activity [35]. In addition, as a catechol, 3-HT possesses similar anti-oxidative properties as other catechols such as quercetin, catechins, and flavonoids. These compounds contain hydroxyl groups and a highly conjugated π-electron system, which allow them to act as free radical scavengers. Flavonoids exert anti-oxidative activity via chelation; hence, they are good protective agents against free radical–mediated cell injury [38].

Previous evidence has revealed that persistent exposure to ROS initiates the pro-apoptotic pathway, resulting in podocyte apoptosis [20]. We further investigated the molecular mechanism of both 3-HT and CDA by analyzing the expression of the pro-apoptotic Bax and the anti-apoptotic Bcl2 proteins. We found no significant difference in the expression of the apoptotic Bax in the PA-treated group. In contrast, Bcl2 was reduced with PA treatment, resulting in a decreased Bcl2/Bax ratio. Therefore, the cells were more susceptible to apoptosis from PA treatment. Our results are similar to a previous study that applied proteomic analysis: PA-treated osteoblasts showed a reduction in Bcl2 expression concomitant with a non-significant increase in Bax expression [39]. We found that a reduction in podocytes correlated with the permeability assay, revealing an increase in FITC-dextran leakage across podocyte monolayers. Interestingly, treatment with 3-HT or CDA upregulated the expression of Bcl2 and reduced FITC-dextran leakage. In contrast to this finding in podocytes, 3-HT showed an anti-proliferative effect via induction of S phase arrest in human ovarian cancer cells [33]. 3-HT also activated the apoptotic pathways by downregulating anti-apoptotic Bcl2 and by activating the cleaved caspase cascade [33]. The difference in the mechanism of 3-HT might depend on each cell type. Although both 3-HT and CDA significantly decreased podocyte death, the protein leakage was still relatively high in the treatment groups compared with the PA-treated group. A possible explanation is that PA may affect the expression or dislocation of proteins required for filtration, such as nephrin. Moreover, PA may alter the cellular structure, which is vital for barrier formation. These alterations could not be restored by 3-HT and CDA.

## 4. Materials and Methods

### 4.1. Cell Line, Chemical Reagents, and Antibodies

The immortalized human podocyte cell line was generated by Professor Moin Saleem (University of Bristol, Bristol, UK) as described previously [40]. Cell culture reagents including RPMI-1640 culture medium (Cat no. 31800089), fetal bovine serum (FBS) (Cat no. 10082147), insulin transferrin selenium (ITS) (Cat no. 41400045), 0.05% trypsin-EDTA (Cat no. 25300062), 100 U/mL penicillin and 100 mg/mL streptomycin (Cat no. 15140122), and Alexa Fluor^TM^ 568 phalloidin (Cat no. A12380), and Hoechst 33342 (Cat no. H3570) were purchased from Thermo Fisher Scientific (Waltham, MA, USA). Reagents for western blotting were purchased from Merck Millipore (Billerica, MA, USA). The anti-Bcl2 antibody (Cat no. ab196495) (Lot: GR249198-86) was purchased from Abcam (Cambridge, MA, USA). The anti-Bax (D2E11) (Cat no. 5023) (Lot: 10) and anti-β-actin (Cat no. 4970S) (Lot: 18) antibodies were purchased from Cell Signaling Technology (Boston, MA, USA). The anti-podocin antibody (Cat no. P0372), sodium palmitate (Cat no. P9767), DPPH (Cat no. D9132), DCFDA (Cat no. D6883), Thiazolyl Blue Tetrazolium Bromide (Cat no. M5655), and FITC-dextran (46945) were purchased from Sigma-Aldrich (Saint Louis, MO, USA). The FITC Annexin V Apoptosis Detection Kit (Cat no. 556547) was purchased from BD Biosciences (Franklin Lakes, NJ, USA). The anti-synaptopodin antibody (Cat no. SC-515842) was purchased from Santa Cruz Biotechnology (Dallas, TX, USA).

### 4.2. Isolation of 3-HT and CDA 

The crude ethyl acetate extract (1.9 g, a dark brown gum) from the mycelia of the marine-derived fungus *A. candidus* PSU-AMF169 was prepared by following a published protocol [41]. It was fractionated by column chromatography (CC) over Sephadex LH-20 using a mixture of methanol (MeOH) and dichloromethane (CH_2_Cl_2_) in a ratio of 1:3 as an eluent to afford 11 fractions (A–K). Fraction H (280.0 mg) was further purified by CC over silica gel with a gradient of MeOH−CH_2_Cl_2_ (4:96→1:0) to afford nine subfractions (H1–H9). Subfraction H3 (13.1 mg) contained CDA, whereas 3-HT (21.3 mg) was obtained by recrystallizing subfraction H5 (186.3 mg) in a mixture of acetone-chloroform. Their structures were determined by using 1D and 2D nuclear magnetic resonance (NMR) spectroscopic data and further confirmed by comparing their NMR data with those previously reported in the literature [42].

### 4.3. Cell Culture

Immortalized human podocytes were cultured as described previously [40]. Briefly, podocytes were cultured in RPMI 1640 medium supplemented with ITS, 10% FBS, 100 U/mL of penicillin, and 100 g/mL streptomycin. The cells were incubated at two different temperatures. For the growth-permissive condition, cells were allowed to proliferate at 33 °C. After the cells reached 80% confluence, they were transferred to a 37 °C incubator (non-permissive condition) to initiate cell differentiation into mature podocytes for 10–14 days before an experiment. Complete differentiation of podocytes was confirmed by expression of podocin and synaptopodin.

### 4.4. Immunofluorescence Staining

Undifferentiated or differentiated podocytes were seeded and grown on glass coverslips for 24 h before fixation with 4% paraformaldehyde for 20 min at room temperature. Cells were then permeabilized with 0.3% Triton X-100 for 15 min and blocked for 1 h with 1% bovine serum albumin (BSA), 0.2% Triton X in phosphate-buffered saline (PBS). Primary antibodies including anti-podocin (1:500) and anti-synaptopodin (1:500) were added and incubated overnight at 4 °C. Then, cells were stained with AlexaFluor™ 488 secondary antibodies (1:500) for 1 h in the dark at room temperature. For F-actin filament staining, cells were stained with Alexa Fluor^TM^ 568 phalloidin (1:1000). Cells were also incubated with Hoechst 33342 (1:1000) for 5 min to stain nuclei. Samples were examined via confocal microscopy.

### 4.5. PA Preparation

PA was prepared as described previously [20]. Sodium palmitate was prepared as a conjugate with BSA. First, sodium palmitate was dissolved in distilled water and heated at 70 °C for 30 min or until the solution became clear. BSA was also dissolved in distilled water and heated at 55 °C for 30 min. Dissolved sodium palmitate was added slowly to the BSA solution. Before an experiment, the PA-BSA solution was diluted in RPMI 1640 cell culture medium to the desired concentrations. The control group was treated with BSA vehicle control.

### 4.6. Cell Viability Assay

Podocytes were seeded on 96-well plates at a density of 2 × 10^4^ cells/well and cultured overnight. Then, cells were cultured for 24 h in RPMI-1640 media containing BSA vehicle control, or PA with or without 3-HT or CDA. The MTT assay was performed to determine cell viability. Briefly, after 24-h treatment, the treatment media were removed and cells were incubated with 0.5 mg/mL MTT in RPMI-1640 media for 2 h in the dark, followed by solution removal and addition of 100 μL dimethyl sulfoxide (DMSO) to dissolve the blue formazan product. Absorbance was measured at 570 nm using a Synergy™ Neo2 Multi-Mode microplate reader (BioTek, Winooski, VT, USA). Data of the percent viable cells at various concentrations of 3-HT or CDA were fit to Hill’s equation to calculate IC_50_ using the Igor Pro 4.07 software.

### 4.7. Flow Cytometric Analysis of Cell Death

For apoptotic cell detection, annexin V-FITC/PI staining was performed according to the instruction manual. Podocytes were seeded on 6-well plates at a density of 2 × 10^5^ cells/well and grown for 24 h before treatment with 600 µM PA in the presence or absence of 3-HT or CDA for 24 h. Cells were washed with cold PBS, trypsinized, centrifuged at 1000 rpm for 5 min, and washed twice with 500 µL of 1X annexin V buffer. After another centrifugation, the cell pellet was resuspended in 100 µL of 1X annexin V buffer. Cells were stained for 15 min with 5 µL of annexin V-FITC, followed by 5 µL of PI in the dark at room temperature, and resuspended in 400 µL of 1X annexin V buffer. Flow cytometry was performed with the Accuri C6 Plus flow cytometer (BD Bioscience, San Diego, CA, USA).

### 4.8. Podocyte Permeability Assay

Differentiated podocytes were seeded overnight at a density of 1.5 × 10^5^ cells/cm^2^ on 1-µm porous membrane culture inserts (MCRP12H48, Merck) coated with collagen type IV at 10 µg/cm^2^. Cells were maintained for 1 week under normal culture conditions unless stated otherwise. On day 6, podocytes were exposed for 24 h to 600 µM PA in the presence or absence of 50 µM 3-HT or CDA.

Transepithelial permeability was determined by measuring fluxes of FITC-dextran (70 kDa) across the podocyte monolayer cultured on inserts. Cells were washed with PBS once before addition of 1.9 mL of 1 mg/mL FITC-dextran solution in PBS (with 5 mM D-glucose, 0.5 mM MgCl_2_, and 0.9 mM CaCl_2_ in the lower compartment and 0.4 mL PBS in the upper compartment. Solutions in the upper compartments were collected after 120 min. The FITC-dextran content and the standard solutions were measured at 485/535 nm using the Synergy™ Neo2 Multi-Mode plate reader (BioTek).

### 4.9. Measurement of ROS Generation

Podocytes were seeded on 96-well plates at a density of 2 × 10^4^ cells/well and grown for 24 h. Podocytes were cultured with RPMI 1640 medium containing BSA vehicle control or 600 µM PA with or without 50 µM 3-HT or CDA for 24 h. Cells were washed twice with PBS, stained with 20 µM DCFDA solution, and incubated for 45 min at 37 °C in the dark. Subsequently, cells were washed twice with PBS, counterstained with Hoechst for 5 min, and washed twice with PBS. Fluorescence intensity was measured at 485/535 nm using the Synergy™ Neo2 Multi-Mode plate reader (BioTek).

### 4.10. Measurement of Scavenging Activity

To evaluate the anti-oxidative activity of 3-HT and CDA, the DPPH free radical scavenging method was used as described previously [43]. DPPH was dissolved in 99% ethanol and kept in the dark for 2 h. DPPH solution (1 mL) was added to a 24-well plate, followed by 200 µL of ethanol, 3-HT, or CDA, and 800 µL of 0.1 M Tris-HCl buffer (pH 7.4). After 30 min of incubation in the dark, absorbance was measured at 517 nm using the Synergy™ Neo2 Multi-Mode plate reader (BioTek).

### 4.11. Western Blot Analysis

Podocytes were seeded on 6-well plates at a density of 2 × 10^5^ cells/well and incubated overnight. Then, cells were incubated for 24 h with FBS-free RPMI 1640 medium containing 10 µM 3-HT or CDA. Proteins were extracted using radioimmunoprecipitation assay (RIPA) lysis buffer containing protease inhibitors (PI), separated using sodium dodecyl sulfate–polyacrylamide gel electrophoresis (SDS-PAGE), and transferred to a nitrocellulose membrane. The membrane was blocked with 5% non-fat dry milk for 1 h and incubated overnight with rabbit anti-Bcl2, anti-Bax, or anti-β-actin antibodies (diluted 1:1000). The membrane was then washed three times with Tris-buffered saline with Tween-20 (TBST) and incubated for 1 h at room temperature with horseradish peroxidase (HRP)–conjugated goat anti-rabbit immunoglobulin G. Protein expression was detected by using the Luminata Forte Western HRP substrate (Merck Millipore, Billerica, MA, USA). Chemiluminescence images were captured by ChemiDoc MP Imaging System (Bio-Rad Laboratories, Inc., Hercules, CA, USA) and analyzed with Image J software (version 1.51).

### 4.12. Statistical Analysis

All results are expressed as the mean ± standard error of the mean. One-way analysis of variance (ANOVA) was used to determine the difference between each group with Tukey’s post hoc test. *p* < 0.05 was considered statistically significant.

## 5. Conclusions

We identified novel biological activity of fungus-derived 3-HT and CDA isolated from the marine-derived fungus *A. candidus* PSU-AMF169 in protecting against PA-induced podocyte apoptosis and barrier dysfunction. Our results indicate that the mechanisms of actions of these two compounds involve direct scavenging of ROS and upregulation of the anti-apoptotic Bcl2 protein. Additional research and development on these classes of compound may provide a novel treatment of DN.

## Figures and Tables

**Table 1:** List of fungi and plants from which the majority of the screened compounds were isolated.

Number	Source	Name of Fungi/Plants
1	Fungi	*Aspergillus* sp.
2	Fungi	*Pestalotiopsis* sp.
3	Fungi	*Trichoderma* sp.
4	Plant	*Desmos* sp.
5	Plant	*Enicosanthum* sp.
6	Plant	*Erythrina* sp.
7	Plant	*Friesodielsia* sp.
8	Plant	*Garcinia* sp.
9	Plant	*Goniothalamus* sp.
10	Plant	*Maclura* sp.
11	Plant	*Melodorum* sp.
12	Plant	*Millettia* sp.
13	Plant	*Mitrephora* sp.
14	Plant	*Murraya* sp.
15	Plant	*Piper* sp.
16	Plant	*Pongamia* sp.
17	Plant	*Uvaria* sp.

## Data Availability

The data presented in this study are available on request from the corresponding author.

## Sample Availability

Samples of the compounds (3-HT and CDA) are available from the authors.
